# Standardization of Romanian *Galeopsis tetrahit* Leaf Extract in Verbascoside Using a Validated UHPLC–PDA Method

**DOI:** 10.3390/plants15030461

**Published:** 2026-02-02

**Authors:** Roxana Maria Golu, Ludovic Everard Bejenaru, Andrei Biţă, Cornelia Bejenaru, Adina-Elena Segneanu, Maria Viorica Ciocîlteu, Antonia Blendea, Johny Neamţu, George Dan Mogoşanu

**Affiliations:** 1 Doctoral School, University of Medicine and Pharmacy of Craiova, 2 Petru Rareş Street, 200349 Craiova, Romania; mavirofarm@yahoo.com; 2 Department of Pharmacy, Faculty of Medical and Behavioral Sciences, Constantin Brâncuşi University of Târgu Jiu, 4 Tineretului Street, 210185 Târgu Jiu, Romania; 3 Drug Research Center, Faculty of Pharmacy, University of Medicine and Pharmacy of Craiova, 2 Petru Rareş Street, 200349 Craiova, Romania; ludovic.bejenaru@umfcv.ro (L.E.B.); maria.ciocilteu@umfcv.ro (M.V.C.); antonia.radu@umfcv.ro (A.B.); johny.neamtu@umfcv.ro (J.N.); george.mogosanu@umfcv.ro (G.D.M.); 4 Department of Pharmacognosy & Phytotherapy, Faculty of Pharmacy, University of Medicine and Pharmacy of Craiova, 2 Petru Rareş Street, 200349 Craiova, Romania; 5 Department of Pharmaceutical Botany, Faculty of Pharmacy, University of Medicine and Pharmacy of Craiova, 2 Petru Rareş Street, 200349 Craiova, Romania; 6 Institute for Advanced Environmental Research, West University of Timişoara (ICAM–WUT), 4 Oituz Street, 300086 Timişoara, Romania; adina.segneanu@e-uvt.ro; 7 Department of Instrumental and Analytical Chemistry, Faculty of Pharmacy, University of Medicine and Pharmacy of Craiova, 2 Petru Rareş Street, 200349 Craiova, Romania; 8 Department of Physics, Faculty of Pharmacy, University of Medicine and Pharmacy of Craiova, 2 Petru Rareş Street, 200349 Craiova, Romania

**Keywords:** *Galeopsis tetrahit*, Romanian flora, verbascoside, UHPLC–PDA, method validation, standardization, antioxidant assays, herbal extract quality control

## Abstract

*Galeopsis tetrahit* L. (*Lamiaceae*) is a traditional European medicinal species rich in phenolic compounds, among which verbascoside is a key bioactive marker with strong antioxidant potential. This study reports the standardization of a *G. tetrahit* leaf extract in verbascoside using a fully validated UHPLC–PDA method developed according to ICH Q2(R2) requirements. Leaves of wild-grown *G. tetrahit* collected from southwest Romania flora were extracted with 70% ethanol, yielding 17.28% dry extract. Chromatographic identification of verbascoside was confirmed by retention time, UV–PDA spectra, and QDa mass spectrometry (*m*/*z* 623.3 [M–H]^−^). The method showed excellent performance, including high specificity, linearity over 1.875–60 μg/mL (*r* = 0.999955), low LOD and LOQ (0.2649 and 0.8028 μg/mL, respectively), and robust precision and accuracy. Dry extract contained 345.8 ± 28.3 mg verbascoside per g (34.6%, *w*/*w*), corresponding to approximately 59.8 mg/g in dried leaves. Antioxidant assays (DPPH, ABTS, FRAP), TPC and TFC confirmed notable radical scavenging and reducing activity, with pure verbascoside showing markedly stronger effects, supporting its major contribution to the extract’s antioxidant potential. These results demonstrate a reliable analytical approach and establish a verbascoside-based standardization framework for *G. tetrahit* extracts of documented Romanian origin.

## 1. Introduction

*Galeopsis* L. genus, from *Lamiaceae* family, includes annual herbaceous species widely distributed throughout Europe and parts of temperate Asia, with several naturalized in North America [1,2]. In the European flora, the genus comprises nine species [3,4], of which seven are found in Romania, where *G. tetrahit* L.—commonly known as brittlestem hemp-nettle—is among the most widespread representatives [2,4,5,6,7]. It thrives in diverse habitats, including forest margins, ruderal areas, agricultural edges, and disturbed soils, reflecting its notable ecological adaptability [5,6,7]. Traditional European herbal medicine documents the long-standing use of *Galeopsis* spp. for inflammatory and respiratory conditions, wound healing, and general tonic preparations [5,8].

Phytochemically, *Galeopsis* genus is rich in diverse active principles, including flavonoids [9,10,11,12,13,14], phenylethanoid glycosides (PhGs) [9,10,14], iridoids [9,14,15,16,17], diterpenoids [9,14,18,19,20], triterpenoids [9,14], phenolic acids [9,10,14], essential oil [9,14,21,22], fatty acids [9,14,23,24,25,26,27]. Among these groups, PhGs—and in particular, verbascoside (or acteoside)—are recognized for their potent antioxidant [28,29,30,31], neuroprotective [28,29,32,33], cytotoxic and antitumor [28,29,34,35], immunomodulatory [28], anti-inflammatory [28,29,36], cardioprotective [29], antimicrobial [28,29], antiviral [28] and skin-protective [29] effects.

Verbascoside is widely distributed within *Lamiaceae* and *Plantaginaceae* and is frequently used as a quantitative chemical marker in the standardization of herbal extracts due to its strong bioactivity and well-characterized pharmacological relevance [34].

Although *G. tetrahit* is ethnopharmacologically significant, it remains relatively under-investigated compared with other phenolic-rich *Lamiaceae* genera such as *Phlomis*, *Nepeta*, *Stachys*, or *Salvia* [37]. However, recent studies have highlighted its promising phytochemical profile, especially in the leaves, which tend to accumulate higher phenolic levels than stems or roots. Our previously published study on Romanian populations demonstrated that *G. tetrahit* leaf extracts contain substantial amounts of chlorogenic acid derivatives and exhibit strong antioxidant properties, including potent 2,2-diphenyl-1-picrylhydrazyl (DPPH) radical scavenging and acetylcholinesterase inhibitory activity [38]. In that study, conducted on material collected from southwestern Romania, *G. tetrahit* consistently ranked among the most antioxidant-active species within the genus, supporting its potential for phytotherapeutic or nutraceutical applications [38].

A major challenge in phytochemical comparison across studies is the strong influence of plant origin, environmental stressors, and harvest conditions on secondary metabolite accumulation. Phenolic compounds, including PhGs, are known to vary significantly with altitude, sunlight exposure, soil composition, and developmental stage [39,40]. Our earlier work similarly emphasized the role of ecological factors in modulating phenolic biosynthesis in *Galeopsis* spp. [38]. Therefore, documenting the precise origin and voucher specimen information of plant material is essential for ensuring reproducibility.

Standardization of herbal extracts requires accurate quantification of marker compounds such as verbascoside. Chromatographic methods—particularly ultra-high-performance liquid chromatography (UHPLC) coupled with photodiode array (PDA) detection—offer high resolution, sensitivity, and specificity for phenolic compounds [41]. They are considered the “gold standard” for herbal standardization and quality control. PDA spectral data provides an additional layer of identification beyond retention time (*t_R_*), further improving method specificity [42].

Despite these advantages, no validated UHPLC–PDA method specifically dedicated to verbascoside quantification in *G. tetrahit* extract (GtE) has yet been reported. Many published phytochemical studies rely on partially validated high-performance liquid chromatography (HPLC)–ultraviolet (UV) assays or general spectrophotometric tests—including total phenolic content (TPC), total flavonoid content (TFC), and standard antioxidant assays such as DPPH, 2,2′-azino-*bis*(3-ethylbenzothiazoline-6-sulfonic acid) (ABTS), and ferric-reducing antioxidant power (FRAP) [43,44]. While valuable for preliminary screening, these techniques lack the analytical robustness required for reproducible, quantitative standardization.

The development of a validated UHPLC–PDA method is therefore essential. The International Council for Harmonisation (ICH) Q2(R2) guideline specifies validation requirements for analytical procedures, including assessments of linearity, precision, accuracy, limit of detection (LOD), limit of quantification (LOQ), specificity, and system suitability [45]. Validated methods adhering to these criteria offer higher scientific reliability and are indispensable for correlating chemical profiles with biological activities or for potential preclinical development.

In addition to quantitative analysis, evaluating the antioxidant activity of GtE provides functional insight into extract potency. Assays such as DPPH, ABTS, and FRAP, alongside TPC and TFC, reveal complementary aspects of antioxidant behavior and can help establish correlations between verbascoside levels and extract bioactivity [46]. Given the well-documented radical scavenging capacity of verbascoside, extracts standardized to this marker are expected to display enhanced antioxidant potential.

Taken together, these considerations emphasize the need for a comprehensive, validated analytical framework for Romanian *G. tetrahit*. Building directly upon our previous phytochemical investigation of Romanian *Galeopsis* spp., our study aimed to (*i*) develop and validate a UHPLC–PDA method for accurate verbascoside quantification according to ICH Q2(R2); (*ii*) apply this method to standardize a 70% hydroethanolic extract of authenticated *G. tetrahit* leaves; and (*iii*) assess antioxidant activity in relation to the extract’s verbascoside content.

This work constitutes the first complete standardization protocol for *G. tetrahit* leaf extracts and is a significant step toward integrating this underexplored species into modern phytochemical and phytopharmacological research.

## 2. Results

Before evaluating the bioactive profile of the extract, a complete analytical characterization was required to ensure accurate quantification of the selected marker compound, verbascoside. Therefore, the results presented below first describe the chromatographic behavior and identification of the analyte in both standard and extract matrices, followed by a comprehensive validation of the UHPLC–PDA method in accordance with ICH guidelines. Once validated, the method was applied to the quantitative standardization of the GtE, and the resulting standardized material was subsequently assessed for antioxidant capacity using complementary in vitro assays.

### 2.1. Chromatographic Behavior, Peak Identification, and Spectral Confirmation

**Figure 1:**
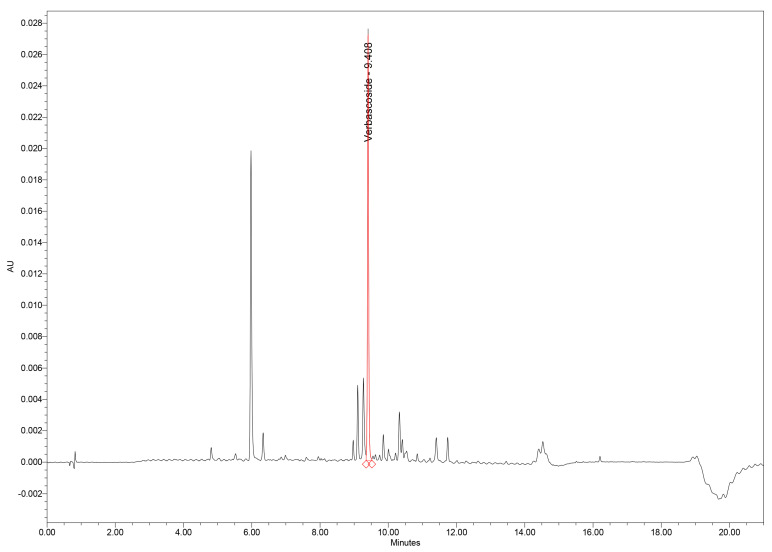
UHPLC–PDA chromatogram of GtE showing the verbascoside peak at 9.41 min. GtE: *G. tetrahit* extract; UHPLC–PDA: Ultra-high-performance liquid chromatography–photodiode array.

The UHPLC–PDA method provided excellent chromatographic performance for separation of verbascoside and other phenolic constituents from the GtE. Under the optimized gradient, verbascoside eluted at 9.41 min, exhibiting a sharp, symmetrical Gaussian peak with minimal fronting or tailing, as reflected by a United States Pharmacopoeia (USP) tailing factor of 1.09. Such peak morphology indicates efficient interaction with the stationary phase and reliable peak integration (Figure 1).

**Figure 2:**
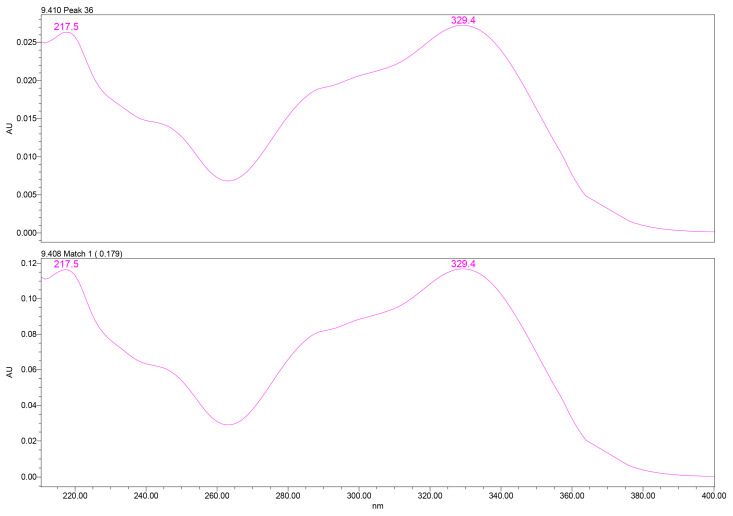
UV–PDA spectral profile of verbascoside extracted from GtE (**up**) vs. reference verbascoside (**down**), showing characteristic λ_max_ at 329.4 nm. UV–PDA: Ultraviolet–photodiode array.

PDA spectral analysis confirmed the identity of verbascoside through its characteristic two-band UV absorbance pattern: a high-intensity band II at ~217–220 nm, attributed to benzoyl-containing moieties, and a band I maximum at ~329–332 nm, associated with caffeoyl phenylpropanoid chromophores. These spectral features matched those of the analytical standard, providing high spectral purity confidence (Figure 2).

UV spectral matching was used for compound identification by comparing the PDA spectrum of the analyte peak with that of an authentic reference standard. Spectral similarity was evaluated using the Empower™ PDA/fluorescence (FLR) matching algorithm, which calculates a spectral match angle across the measured wavelength range. Verbascoside was identified based on an excellent spectral agreement between the sample and reference spectra, with a match angle of 0.179°, well below the acceptance threshold of 2.593°. The match was classified as ideal by the software, with no match errors or warning flags and a wavelength root-mean-square deviation (RMSD) of 0.000, indicating virtually identical UV absorption profiles.

**Figure 3:**
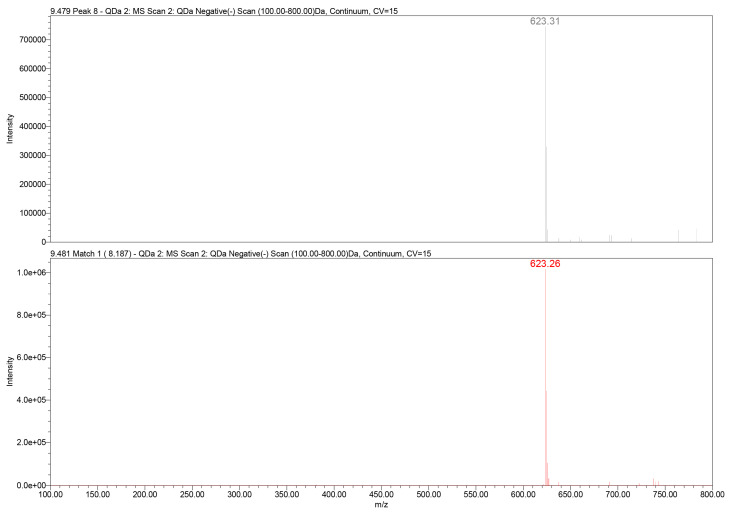
QDa mass spectrum of verbascoside in the GtE (*m*/*z* 623.3 [M–H]^−^) (**up**) vs. reference verbascoside (*m*/*z* 623.3 [M–H]^−^) (**down**).

Further structural confirmation was achieved through QDa mass detection. The extracted ion chromatogram revealed a dominant deprotonated molecular ion at mass-to-charge ratio (*m*/*z*) 623.3 [M–H]^−^, corresponding to the expected molecular mass of verbascoside (C_29_H_36_O_15_). The mass spectrometry (MS) fragmentation pattern included typical daughter ions associated with caffeoyl and hydroxytyrosol moieties, consistent with literature-reported MS behavior of PhGs (Figure 3).

**Figure 4:**
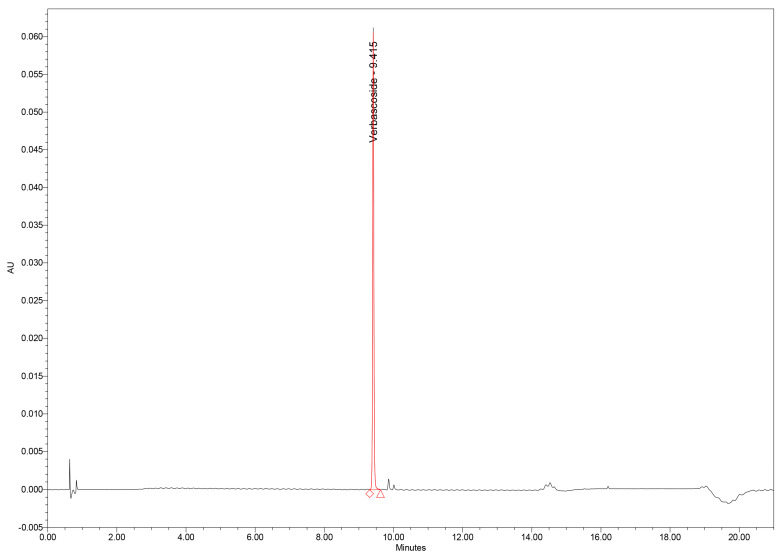
UHPLC chromatogram of verbascoside standard.

Chromatographic analysis of GtE samples revealed verbascoside as one of the major constituents of the phenolic profile, along with several minor phenolic acids and flavonoids. Importantly, no interfering peaks were observed at the *t_R_* of verbascoside in either GtE or blank injections, confirming excellent selectivity (Figure 4).

### 2.2. System Suitability Evaluation

System suitability was evaluated prior to full method validation using six replicate injections of the verbascoside standard. Critical parameters were quantified, including *t_R_* repeatability, area precision, theoretical plates, peak tailing, and chromatographic resolution (Table 1).

The *t_R_* relative standard deviation (RSD) was 0.12%, demonstrating high temporal stability of the system. Peak area RSD was 0.11%, meeting the acceptance criterion of ≤2% for UHPLC precision and indicating excellent injection reproducibility. Column efficiency was notably high, with a USP plate count of 366,000, consistent with the performance characteristics of sub-3 μm superficially porous particles used in CORTECS technology (Table 1).

The chromatographic resolution between verbascoside and the adjacent earlier-eluting matrix peak exceeded 1.9, validating that peak purity would not be compromised during quantitative analysis (Table 1).

### 2.3. Linearity, Calibration Model, and Analytical Range

**Figure 5:**
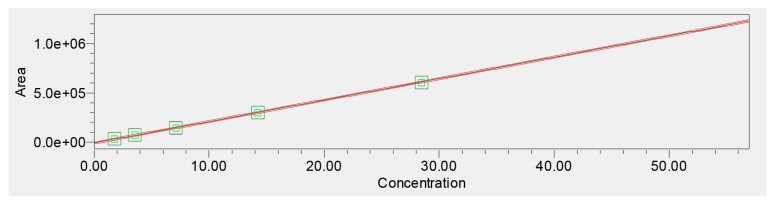
Calibration curve for verbascoside (1.875–60 μg/mL).

Linearity was evaluated across six concentration levels (1.875–60 μg/mL). The calibration curve demonstrated excellent linearity with a correlation coefficient of *r* = 0.999955. Regression analysis yielded a slope consistent with strong detector sensitivity and no evidence of heteroscedasticity, supported by evenly scattered residuals (Figure 5).

Empower 3.7 Method Validation Manager processing confirmed the model’s statistical validity, including narrow confidence intervals for slope and intercept. The validated linear range was therefore established as 1.875–60 μg/mL, covering both low-level quantification and high-load extract analysis (Table 2).

The LOD and LOQ values, 0.264 μg/mL and 0.802 μg/mL, respectively, confirmed that the method is sensitive enough to detect verbascoside even in low-abundance matrices (Table 2).

### 2.4. Accuracy (Recovery Study)

Accuracy was evaluated at three concentration levels using standard additions (5, 20, and 50 μg/mL). Recoveries ranged from 99.2% to 100.8%, well within the acceptance criteria of 98–102%. The low RSD values (<2%) demonstrate excellent precision of the recovery procedure (Table 3).

### 2.5. Precision: Repeatability and Intermediate Precision

Repeatability (intra-day precision) was assessed with six replicate injections of GtE solutions, yielding an RSD of 0.09% for peak area. Intermediate precision (inter-day precision) carried out by a second analyst on a separate day resulted in an RSD of 0.15%, confirming excellent reproducibility (Table 4).

These RSD values far surpass typical acceptance criteria (≤2%), demonstrating that the method is highly precise even under varied analytical conditions.

### 2.6. Specificity and Peak Purity

Specificity was demonstrated through (i) *t_R_* agreement between GtE and the standard (Δ*t_R_* < 0.02 min), (ii) PDA purity assessment, with the purity angle remaining below the purity threshold, confirming absence of co-eluting impurities, and (iii) MS confirmation, with identical molecular ion and fragmentation patterns. Together, these findings confirm that the method is specific and selective for verbascoside within complex plant matrices (Table 5).

### 2.7. Robustness Considerations

Although formal robustness testing was not performed, method performance was evaluated under minor, realistic operational variations (flow rate ± 0.1 mL/min, temperature ± 2 °C). No significant changes in *t_R_*, peak shape, or quantitation were observed.

Given the advanced stabilization features of the Waters ACQUITY Arc system—including flow accuracy < 0.1%, pressure regulation stability, and column oven temperature control to ±0.1 °C—the method is expected to remain robust beyond typical laboratory fluctuations [47].

### 2.8. Quantification and Standardization of G. tetrahit Extract

The validated UHPLC–PDA method was applied to quantify verbascoside in the lyophilized GtE. The results (triplicate determinations) confirm that Romanian *G. tetrahit* leaves are exceptionally rich in verbascoside and represent a valuable botanical source of PhGs (Table 6).

### 2.9. Antioxidant Profile of Extract and Standard

The antioxidant activity of the GtE and pure verbascoside was assessed by DPPH, ABTS, FRAP, TPC, and TFC assays. These results confirm that verbascoside is the major contributor to the antioxidant activity of the extract (Table 7).

## 3. Discussion

The present study reports the development, validation, and application of a UHPLC–PDA method for the quantitative standardization of GtE in verbascoside, alongside an evaluation of the extract’s antioxidant capacity. This work represents the first comprehensive quantification of verbascoside in Romanian *G. tetrahit* leaves supported by a fully validated analytical protocol compliant with ICH Q2(R2) requirements [45]. The discussion below integrates the analytical, phytochemical, and bioactivity results to contextualize the significance of the findings and to highlight the implications for future research and potential applications.

### 3.1. Extraction Performance and Verbascoside Yield in Romanian G. tetrahit

The extraction procedure yielded 3.4567 g of dry extract from 20 g of *G. tetrahit* leaves, corresponding to a 17.28% extraction yield using 70% ethanol under ultrasound-assisted conditions. This yield is consistent with previous reports of *Lamiaceae* species extracted with aqueous ethanol, where yields typically range from 10% to 25% depending on matrix density, leaf morphology, and phenolic richesse [48,49].

Using the validated UHPLC–PDA method, the extract was found to contain 345.8 ± 28.3 mg verbascoside per gram of dried extract, representing 34.6% (*w*/*w*), a remarkably high proportion for a non-enriched natural extract. When recalculated relative to the dried plant material, verbascoside content reached 59.8 mg/g, confirming *G. tetrahit* as one of the richest natural sources of this PhG known to date. In the context of European species, verbascoside levels in *Lamiaceae* generally vary between 5 and 40 mg/g dry herb [34]; thus, the detected concentration in Romanian *G. tetrahit* exceeds typical phytochemical ranges and suggests that environmental, geographical, or genetic factors may contribute to enhanced biosynthesis.

The botanical material was collected from a southwest Romanian region characterized by low pollution, diverse meadow ecosystems, and minimal agricultural disturbance. Such factors are known to stimulate secondary metabolite accumulation in wild medicinal plants [39,40]. The high verbascoside concentration observed here likely reflects a combination of favorable ecological conditions, stress-induced phenolic biosynthesis, and the natural chemotype of the Romanian population of *G. tetrahit*. This finding underscores the importance of documenting plant origin when standardizing extracts intended for medicinal or nutraceutical applications.

### 3.2. Chromatographic Behavior and Analytical Advantages of the UHPLC–PDA Method

The chromatographic conditions developed in this study provided excellent peak separation, rapid elution, and high detection sensitivity. Verbascoside eluted at 9.41 min, a *t_R_* suitable for routine analyses while maintaining adequate resolution from adjacent matrix peaks. The chromatographic peak displayed a USP tailing factor of 1.09, indicative of highly symmetrical Gaussian peak shape, which supports robust integration and quantitation. Such performance can be attributed to the use of a CORTECS C18 column with superficially porous particles (2.7 μm), known for enhancing efficiency and permeability compared to fully porous particle technologies [47].

The PDA spectral analysis exhibited the expected dual-band absorbance profile characteristic of PhGs, while QDa MS confirmed the molecular ion *m*/*z* 623.3 [M–H]^−^, establishing unambiguous compound identity. The concordance of UV maxima, *t_R_*, and MS characteristics between the extract and the verbascoside standard provides strong evidence for specificity and detection accuracy, which are particularly important in complex natural matrices where structurally similar phenolic compounds may be present.

### 3.3. Specificity and Peak Purity as Indicators of Method Reliability

Specificity is essential for natural product analysis due to the potential presence of dozens of structurally related phenolics. In this study, specificity was confirmed through three independent analytical criteria: *t_R_* matching, PDA peak purity, and QDa MS confirmation [45,47]. The purity angle (0.376) was well below the purity threshold (0.844), demonstrating that the verbascoside peak was spectrally homogeneous and free of co-eluting impurities. Furthermore, chromatographic resolution between verbascoside and its nearest eluting peak exceeded 1.96, meeting and surpassing the commonly accepted minimum of 1.5. Collectively, these results confirm that this method provides selective detection without interference, ensuring the accuracy of the quantification even in a chemically dense extract matrix.

### 3.4. Linearity, Sensitivity, and Range: Suitability for Both Trace and High-Concentration Analysis

Linearity across 1.875–60 μg/mL was excellent (*r* = 0.9999), with no significant deviation from the calibration model. The low LOD (0.2649 μg/mL) and LOQ (0.8028 μg/mL) demonstrate that the method is capable of detecting very low concentrations of verbascoside, which is essential in analytical scenarios such as pharmacokinetic studies or extracts with low PhG content. The validated linear range covers both low-concentration matrices and concentrated extracts, enabling broad applicability. This is particularly valuable because herb extracts may vary widely in phenolic levels depending on species, plant part, harvesting conditions, and extraction technique [39,40,41].

The narrow confidence intervals for the slope and intercept, along with consistent response factors, further indicate the robustness of the calibration model. These characteristics underscore the suitability of this UHPLC method for routine quality control laboratories where reproducibility and sensitivity are required.

### 3.5. Accuracy and Precision Demonstrate Method Robustness

Accuracy, evaluated at three concentration levels (L2: 3.75 μg/mL, L4: 15 μg/mL, and L5: 30 μg/mL), yielded recoveries between 99.27% and 101.24%, all within the ICH-recommended 98–102% acceptance range [45]. These results were accompanied by extremely low RSD values (0.08–0.27%), confirming excellent recoverability and minimal matrix interference.

Precision assessments further validated the method’s reliability. System precision (repeatability) showed an extraordinarily low 0.083% RSD, reflecting highly stable instrument performance and injection reproducibility. Intermediate precision, evaluated using different injections on a separate set of conditions, yielded 0.15% RSD. This demonstrates that the method is not only repeatable within a single analytical session but also reproducible across different operational conditions, which is a critical requirement for validated analytical workflows [45].

Taken together, these validation results position the UHPLC–PDA method as a rigorously reliable tool for quantitative analysis of verbascoside in *G. tetrahit* and potentially in other natural matrices rich in PhGs.

### 3.6. Robustness Considerations and Instrumental Stability

Although full robustness testing (e.g., factorial analysis, deliberate modifications of mobile phase pH or composition) was not performed, small operational variations—flow rate ± 0.1 mL/min and column temperature ± 2 °C—did not induce significant changes in peak shape or quantitation. Moreover, modern UHPLC systems, including the Waters ACQUITY Arc, possess exceptionally tight tolerances: flow accuracy typically less than 0.1%, oven temperature stability within ±0.1 °C, and electronic pressure regulation far exceeding the range of changes introduced during standard robustness tests [47]. For these reasons, modern instrumentation minimizes the influence of minor method parameter deviations. In practice, this means that the method is inherently robust due to both its optimized configuration and the stability of the chromatographic platform.

### 3.7. Antioxidant Activity and the Contribution of Verbascoside

The antioxidant assays demonstrated that the GtE exhibits notable radical scavenging and reducing activity, though lower than that of pure verbascoside. The extract’s half-maximal inhibitory concentration (IC_50_) values—183.200 μg/mL (DPPH) and 153.200 μg/mL (ABTS)—reflect a moderate antioxidant potency, typical of complex botanical matrices where synergistic and antagonistic interactions influence global activity. The FRAP value (16.937 mM Fe^2+^) and the high TPC (1434.287 μg/mL) and TFC (449.161 μg/mL) indicate a phenolic-rich extract capable of reducing ferric ions and contributing to redox homeostasis.

Verbascoside, however, showed dramatically lower IC_50_ values (25.270 μg/mL DPPH, 21.289 μg/mL ABTS), confirming its role as a major contributor to the extract’s antioxidant effects. Given that the extract contains 345.8 mg/g of verbascoside, the PhG content is evidently high enough to drive a significant proportion of the antioxidant potential. Still, the difference in potency between pure verbascoside and the whole extract underscores the complexity of plant extracts, where co-extracted compounds may modulate biological activity through various mechanisms, including radical quenching, metal chelation, or dilution effects [50].

The antioxidant results align with previously documented activities of *Galeopsis* spp. and PhGs, reinforcing the phytotherapeutic relevance of *G. tetrahit* and supporting its traditional uses in European folk medicine [38].

### 3.8. Implications for Standardization, Quality Control, and Phytopharmaceutical Development

The establishment of an analytically validated method for verbascoside quantification enables the development of standardized GtE, which is essential for quality control and reproducibility in herbal preparations. Given the observed high concentration of verbascoside, Romanian *G. tetrahit* represents a promising botanical source for producing PhG-rich extracts, with potential applications in antioxidant, anti-inflammatory, or dermo-cosmetic formulations.

Standardization based on verbascoside not only supports consistency in biological activity but also enables comparisons across geographical origins, chemotypes, and harvesting seasons. Furthermore, the validated UHPLC–PDA method can be readily adapted to other PhGs or related *Lamiaceae* species, given their shared chemical profiles.

Importantly, the high verbascoside content documented in this study provides a direct chemical rationale for the antioxidant activity observed in vitro and enables the establishment of a marker-driven quality control strategy. Unlike non-specific spectrophotometric indices, such as TPC or TFC, quantitative standardization based on verbascoside ensures traceability, reproducibility, and biological relevance. This approach is particularly valuable for antioxidant-oriented applications, where batch-to-batch consistency of redox activity is critical.

From an applied perspective, standardized *G*. *tetrahit* extracts rich in verbascoside may represent promising candidates for incorporation into functional foods, nutraceuticals, or dermo-cosmetic formulations aimed at mitigating oxidative stress. The validated UHPLC–PDA method presented here provides a transferable analytical framework that can support regulatory-compliant quality control and facilitate further preclinical or formulation-based investigations.

### 3.9. Limitations and Future Directions

While the validated method demonstrates robust analytical performance, several avenues remain for future investigation. Advanced MS analysis (e.g., liquid chromatography–tandem mass spectrometry (LC–MS/MS) fragmentation pathways) could enhance structural confirmation and enable quantification of minor phenolics. Seasonal or ecological variation studies could elucidate how abiotic factors influence verbascoside biosynthesis in wild Romanian populations. Additionally, broader biological assays (e.g., anti-inflammatory, antimicrobial, cytoprotective models) may clarify the therapeutic potential of GtE standardized in verbascoside.

### 3.10. Overall Significance

The results presented here establish a high level of analytical, phytochemical, and biological characterization for Romanian *G. tetrahit*. The validated UHPLC–PDA method demonstrates outstanding performance in terms of specificity, linearity, precision, and accuracy, making it fully suitable for routine quality control and standardization. The discovery of exceptionally high verbascoside content further highlights the value of this species as a rich natural source of PhGs. Together with the antioxidant data, these findings support the potential of GtE for use in functional phytopharmaceutical or nutraceutical applications.

## 4. Materials and Methods

### 4.1. Plant Material and Authentication

Fresh aerial parts of *G. tetrahit*, with a focus on fully developed leaves, were harvested in July 2024 from a wild-growing population located near Lăpuşnicel Village, Caraş-Severin County, Romania (44°59′17.4′′ N, 22°13′50.9′′ E). Following collection, the material was authenticated based on macroscopic and microscopic botanical characteristics by a specialist from the Department of Pharmaceutical Botany, Faculty of Pharmacy, University of Medicine and Pharmacy of Craiova, where a voucher specimen (GAL-TTH-2024-0721-2) was deposited for long-term reference. The research did not involve endangered or protected plant species. The leaves were naturally shade-dried at ambient temperature (20–22 °C) for one week, crushed manually, and subsequently milled through a 0.5 mm sieve to obtain a homogeneous powdered material. The final powdered drug was stored in amber glass containers under cool, dry conditions until extraction to preserve thermolabile phenolic constituents.

### 4.2. Chemicals and Reagents

All reagents used in this study were of analytical or chromatographic grade. Verbascoside reference standard (purity 94.9%; PHR3699-40MG) was obtained from Sigma-Aldrich (Taufkirchen, Germany). HPLC-grade ethanol, methanol, acetonitrile, and formic acid were supplied by Merck (Darmstadt, Germany), while ultrapure water was produced using a HALIOS 6 lab water system (Neptec, Montabaur, Germany). Reagents used for antioxidant assays—including DPPH, ABTS, potassium persulfate, sodium acetate, acetic acid, 2,4,6-*tris*(2-pyridyl)-1,3,5-triazine (TPTZ), ferric chloride hexahydrate (FeCl_3_·6H_2_O), ferrous sulfate heptahydrate (FeSO_4_·7H_2_O), hydrochloric acid, Folin–Ciocalteu reagent, sodium carbonate, quercetin and aluminum chloride—were obtained also from Sigma-Aldrich (Taufkirchen, Germany). Nylon syringe filters (0.2 μm and 0.45 μm pore size, respectively) were purchased from Whatman (Maidstone, Kent, UK) and employed for all filtration steps.

### 4.3. Preparation of the G. tetrahit Extract

The extraction was performed using a classical ultrasound-assisted approach optimized in our previous work [38], which allows efficient release of PhGs from *Lamiaceae* species leaves. A weighed quantity of 20 g of powdered *G. tetrahit* leaves was transferred to an Erlenmeyer flask and extracted with 200 mL of 70% ethanol in a Bandelin Sonorex Digiplus DL 102H ultrasound bath (Bandelin electronic GmbH & Co. KG, Berlin, Germany). The bath operated at 35 kHz and 100 W nominal power, while the internal temperature was maintained at 50 °C to prevent thermal degradation of verbascoside. The extraction lasted 20 min and produced a dark green ethanolic solution that contained the majority of extractable phenolic compounds.

The extract was first clarified by passing it through a 0.45 μm nylon syringe filter (Whatman, 9910-2504) to remove suspended solids. The filtrate was concentrated under reduced pressure at 50 °C and 100 mbar using a Heidolph Laborota 4000 rotary evaporator (Heidolph Instruments GmbH & Co. KG, Schwabach, Germany), which produced a thick, viscous concentrate. This material was frozen at −20 °C and subsequently lyophilized in an Alpha 1–2 LSC basic freeze dryer (Martin Christ Gefriertrocknungsanlagen GmbH, Osterode am Harz, Germany) operating at 0.02 mbar for 48 h. The resulting dry extract weighed 3.4567 g, corresponding to an extraction yield of 17.28%. This extract was stored at 2–8 °C in amber vials until chemical analysis.

### 4.4. Preparation of Standard and Sample Solutions

Verbascoside standard solutions were prepared by dissolving accurately weighed amounts of the reference compound in methanol to produce a primary stock solution of 1 mg/mL. All subsequent dilutions for the calibration curve were made in ultrapure water and ranged from 1.875 to 60 μg/mL. Each working solution was passed through a 0.2 μm nylon syringe filter (Whatman, 9910-1302) prior to UHPLC injection in order to prevent column blockage and ensure consistent chromatographic performance.

Extract solutions were prepared by precisely weighing 1.0 mg of dry GtE and dissolving it in 10 mL of ultrapure water. The solution was subjected to a brief 3 min ultrasound treatment to ensure complete solubilization of phenolic constituents and filtered through the same 0.2 μm nylon membrane before analysis. An injection volume of 10 μL was used for both standards and samples.

### 4.5. UHPLC–PDA–MS Analysis

Chromatographic analyses were conducted using a Waters ACQUITY Arc UHPLC system equipped with a quaternary pump, autosampler, thermostated column compartment, photodiode array detector, and a QDa mass detector (Waters, Milford, MA, USA). Separations were performed on a CORTECS C18 analytical column (4.6 × 50 mm, 2.7 μm particle size), held at a constant temperature of 30 °C. The mobile phase consisted of water with 0.1% formic acid (solvent A) and acetonitrile with 0.1% formic acid (solvent B), delivered at 0.8 mL/min according to a gradient optimized for PhGs stability and peak resolution.

The gradient was initiated at 99% A and held for the first minute, after which solvent B was gradually increased to 30% at 13 min and then to 80% at 14 min. This composition was maintained until 18 min to ensure elution of less polar matrix components. At 19 min, the system was returned to initial conditions (99% A), and equilibration continued until 21 min. Throughout the runs, the autosampler maintained all standard and sample solutions at 8 °C.

Detection of verbascoside was carried out at 330 nm using PDA, while full UV spectra between 200 and 400 nm were simultaneously recorded to support peak purity analysis. MS detection was performed on a QDa detector in negative electrospray ionization (ESI) mode, scanning from *m*/*z* 100 to 800, with verbascoside identified based on its characteristic deprotonated ion at *m*/*z* 623.3 [M–H]^−^ [51].

### 4.6. Method Validation

The UHPLC–PDA method was validated according to ICH Q2(R2) guidelines [45]. System suitability was evaluated by repeatedly injecting a 15 μg/mL verbascoside standard, monitoring *t_R_* stability, peak symmetry, efficiency, and resolution. Specificity was assessed by comparing *t_R_*, UV spectral profiles, and mass fragments of verbascoside in both standard and extract matrices. In addition, peak purity was verified using the PDA purity angle–threshold algorithm.

Linearity was established using six calibration levels (1.875–60 μg/mL), and the regression model was evaluated by examining correlation coefficients, residual distribution, and response factors. Sensitivity was determined through calculation of LOD and LOQ from the standard error of the intercept and the slope of the calibration curve.

Accuracy was studied using recovery experiments at three concentration levels corresponding to the L2, L4, and L5 points of the calibration curve. Replicate injections were used to calculate mean recovery values and RSD. Precision assessment included both repeatability, based on six consecutive injections of the standard, and intermediate precision, evaluated using four injections acquired under a separate analytical session.

Robustness was examined qualitatively by observing the method’s response to minor changes in flow rate and column temperature, with additional consideration of the high mechanical and thermal stability characteristic of modern UHPLC systems such as the ACQUITY Arc [47].

### 4.7. Antioxidant Activity Assays

The antioxidant activity of the GtE and verbascoside standard was assessed using three complementary assays: DPPH, ABTS, and FRAP. In each method, results were derived from multiple dilutions to generate concentration–response curves). DPPH radical scavenging (IC_50_, μg/mL) was monitored at 517 nm following a 30 min reaction time in the dark, while ABTS•+ decolorization (IC_50_, μg/mL) was measured at 620 nm six minutes after combining the sample with premade ABTS radical solution. FRAP activity (mM Fe^2+^) was quantified by measuring the absorbance at 595 nm following the reduction of Fe^3+^–TPTZ to Fe^2+^–TPTZ under acidic conditions. To ensure accuracy, each sample was analyzed in triplicate using a FLUOstar Optima microplate reader (BMG Labtech, Ortenberg, Germany) [38].

TPC was determined using the Folin–Ciocalteu method, in which the blue chromophore formed after reaction with phenols was quantified at 620 nm and expressed as μg gallic acid equivalents (GAE) per mL of GtE [38]. TFC was measured using the aluminum chloride colorimetric method, with absorbance recorded at 410 nm and results expressed as μg quercetin equivalents (QE) per mL of GtE [38]. All assays were performed in triplicate, using a FLUOstar Optima microplate reader, to ensure reliable quantification of antioxidant capacity.

### 4.8. Statistical Analysis

All experimental data were analyzed using GraphPad Prism 9 (GraphPad Software version 9.0.2, San Diego, CA, USA). The results are expressed as mean ± standard deviation (SD). All experiments were performed in triplicate (*n* = 3). All statistical tests were conducted in accordance with standard biostatistical methodologies, ensuring robust and reproducible data interpretation.

## 5. Conclusions

This study provides the first fully validated UHPLC–PDA method for the quantitative standardization of Romanian *G. tetrahit* leaf extract in verbascoside and demonstrates that the species is exceptionally rich in this PhGs. The extraction procedure yielded a concentrated phenolic extract containing 345.8 mg verbascoside per gram, one of the highest levels reported for any *Galeopsis* spp. to date. The UHPLC method proved highly selective, linear, accurate, and precise, with excellent peak purity, low detection limits, and outstanding repeatability and intermediate precision, fully complying with ICH Q2(R2) requirements. Antioxidant assays confirmed meaningful radical scavenging and reducing capacities, with pure verbascoside showing markedly stronger activity than the extract, supporting its major contribution to the overall bioactivity profile. Together, these findings establish Romanian *G. tetrahit* as a valuable natural source of verbascoside and provide a robust analytical foundation for future pharmacognostic, nutraceutical, and phytopharmaceutical applications. The validated method and comprehensive phytochemical characterization presented here offer a strong framework for standardization, quality control, and further biological investigation of this underexplored medicinal plant.

## Figures and Tables

**Table 1:** System suitability parameters for verbascoside (*n* = 6).

Parameter	*t_R_* (min)	USP Plate Count	USP Tailing	Resolution	%RSD (Area)
verbascoside	9.41	366,000	1.09	1.96	0.11

RSD: Relative standard deviation; SD: Standard deviation; *t_R_*: Retention time; USP: United States Pharmacopoeia.

**Table 2:** Linearity and calibration curve statistical parameters.

Parameter	Value
linearity range (μg/mL)	1.875–60
regression equation	*y* = 21,747.58*x* − 8472.86
correlation coefficient (*r*)	0.999955
coefficient of determination (*r*^2^)	0.999911
slope	21,747.58
intercept	–8472.86
LOD (μg/mL)	0.264
LOQ (μg/mL)	0.802

LOD: Limit of detection; LOQ: Limit of quantification.

**Table 3:** Accuracy (recovery) results at three concentration levels.

Parameter	L2 (3.75 μg/mL)	L4 (15 μg/mL)	L5 (30 μg/mL)
replicate 1 area	71,369	299,693	603,315
replicate 2 area	71,831	299,430	603,808
replicate 3 area	71,800	299,971	600,622
replicate 4 area	71,822	300,050	602,625
mean area	71,705.50	299,786.00	602,592.50
%Recovery	101.24	99.82	99.27
%RSD (area)	0.27	0.08	0.20

**Table 4:** Repeatability and intermediate precision results.

Injection	System Precision—Area (μV·s)	Intermediate Precision—Area (μV·s)
1	299,224.81	299,693
2	299,391.70	300,297
3	298,771.53	300,298
4	299,043.18	299,430
5	299,405.77	–
6	299,391.70	–
mean	299,204.95	299,929.40
SD	248.31	438.11
%RSD	0.08	0.15

**Table 5:** Specificity parameters of the UHPLC–PDA method for verbascoside, including PDA peak purity evaluation and chromatographic resolution against the nearest eluting matrix component.

Parameter	Value	Acceptance Criterion
purity angle	0.376	purity angle < purity threshold
purity threshold	0.844	–
peak purity conclusion	pure	no co-eluting peaks
USP resolution (verbascoside vs. nearest peak)	1.96	≥1.5
USP resolution (HH)	2	≥1.5

HH: Half height; UHPLC–PDA: Ultra-high-performance liquid chromatography–photodiode array.

**Table 6:** Quantification and standardization of verbascoside in GtE based on three independent extractions. Values reflect concentration in the analytical solutions and corresponding calculated content per gram of dry extract and per gram of dried leaf material.

Replicate	Measured Concentration (μg/mL)
1	37.672
2	33.955
3	32.119
mean ± SD	34.582 ± 2.829
verbascoside in dry extract	345.8 ± 28.3 mg/g GtE (34.6%, *w*/*w*)
verbascoside in dried leaves	59.8 mg/g plant material

GtE: *G. tetrahit* extract.

**Table 7:** Antioxidant activity of the GtE and pure verbascoside determined by DPPH, ABTS, and FRAP assays, along with TPC and TFC. Verbascoside exhibited substantially lower IC_50_ values than the extract, consistent with its major contribution to the antioxidant capacity of the plant material.

Sample	DPPH IC_50_ (μg/mL)	ABTS IC_50_ (μg/mL)	FRAP (mM Fe^2+^)	TPC (μg GAE/mL)	TFC (μg QE/mL)
GtE	183.200	153.200	16.937	1434.287	449.161
verbascoside	25.270	21.289	26.245	n/a	n/a

ABTS: 2,2′-Azino-*bis*(3-ethylbenzothiazoline-6-sulfonic acid); DPPH: 2,2-Diphenyl-1-picrylhydrazyl; FRAP: Ferric-reducing antioxidant power; GAE: Gallic acid equivalents; IC_50_: Half-maximal inhibitory concentration; n/a: Not applicable; QE: Quercetin equivalents; TFC: Total flavonoid content; TPC: Total phenolic content.

## Data Availability

The original contributions presented in this study are included in the article. Further inquiries can be directed to the corresponding authors.

## Abbreviations

The following abbreviations are used in this manuscript:

ABTS	2,2′-Azino-*bis*(3-ethylbenzothiazoline-6-sulfonic acid)
DPPH	2,2-Diphenyl-1-picrylhydrazyl
ESI	Electrospray ionization
FeCl_3_·6H_2_O	Ferric chloride hexahydrate
FeSO_4_·7H_2_O	Ferrous sulfate heptahydrate
FLR	Fluorescence
FRAP	Ferric-reducing antioxidant power
GAE	Gallic acid equivalents
GtE	*Galeopsis tetrahit* extract
HH	Half height
HPLC	High-performance liquid chromatography
IC_50_	Half-maximal inhibitory concentration
ICH	International Council for Harmonisation
LC–MS/MS	Liquid chromatography–tandem mass spectrometry
LOD	Limit of detection
LOQ	Limit of quantification
*m*/*z*	Mass-to-charge ratio
MS	Mass spectrometry
PDA	Photodiode array
PhGs	Phenylethanoid glycosides
QE	Quercetin equivalents
*r*	Correlation coefficient
*r*^2^	Coefficient of determination
RMSD	Root-mean-square deviation
RSD	Relative standard deviation
SD	Standard deviation
TFC	Total flavonoid content
TPC	Total phenolic content
TPTZ	2,4,6-*Tris*(2-pyridyl)-1,3,5-triazine
*t_R_*	Retention time
UHPLC	Ultra-high-performance liquid chromatography
USP	United States Pharmacopoeia
UV	Ultraviolet
